# Supplementary figures and images for: pH-dependent activation of cytokinesis modulates Escherichia coli cell size
Source: PLoS Genet. 2020 Mar 23;16(3):e1008685. doi: 10.1371/journal.pgen.1008685 (PMC7117782; doi:10.1371/journal.pgen.1008685)

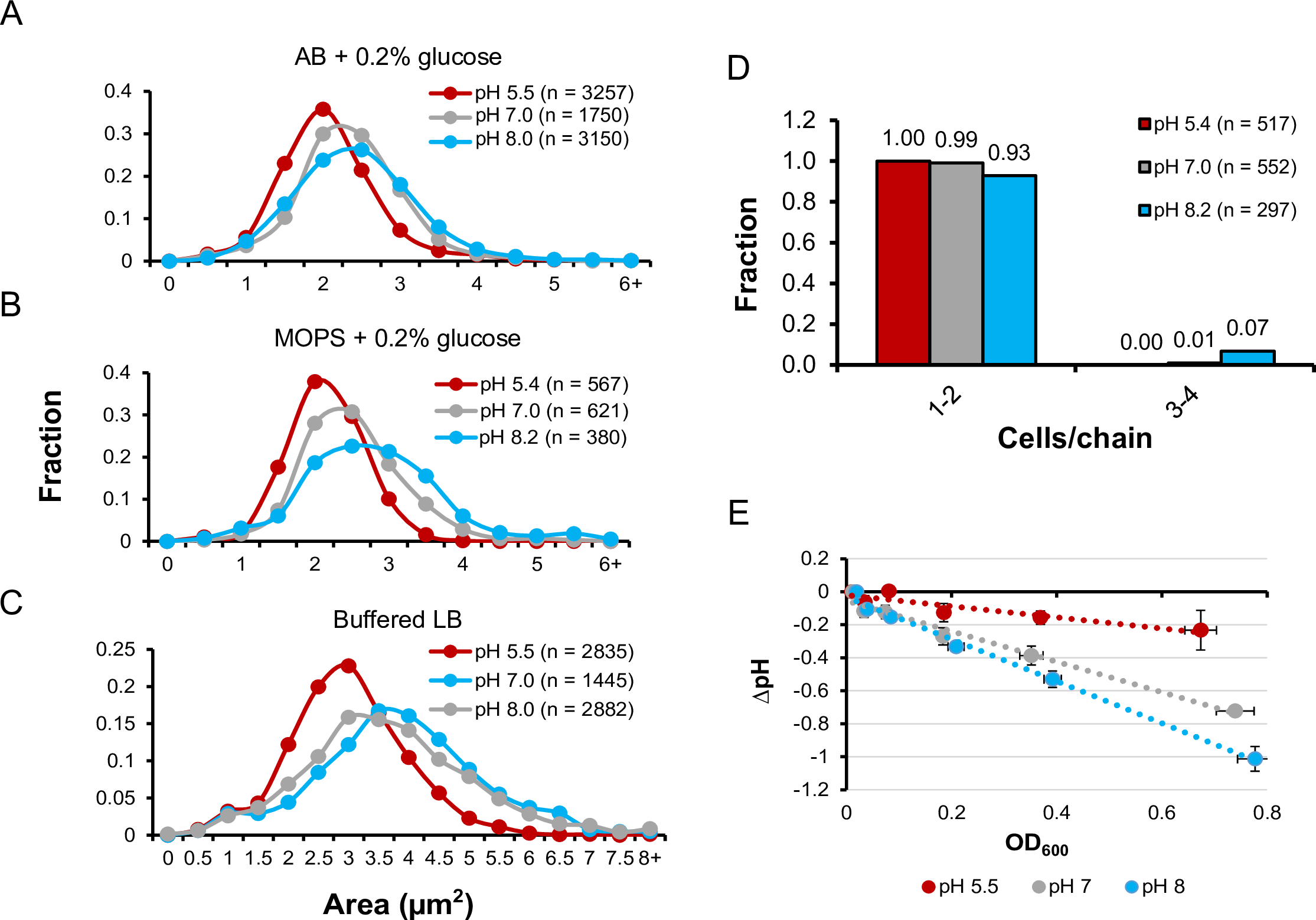

Supplement: S1 Fig — A-C) Cell area distribution of MG1655 grown to steady state in AB minimal medium + 0.2% glucose (A), MOPS minimal medium + 0.2% glucose (B), or LB medium supplemented with 100 mM MES (pH 5.5) or HEPES (pH 7.0 or pH 8.0) (C) and collected for imaging at OD600 ~ 0.1–0.2. D) Fraction of cells present in chains as a function of medium pH during growth in MOPS minimal medium + 0.2% glucose. E) Change in pH as a function of optical density in unbuffered LB medium. Cells were inoculated at an OD600 = 0.005. (TIF) [file pgen.1008685.s001.tif]

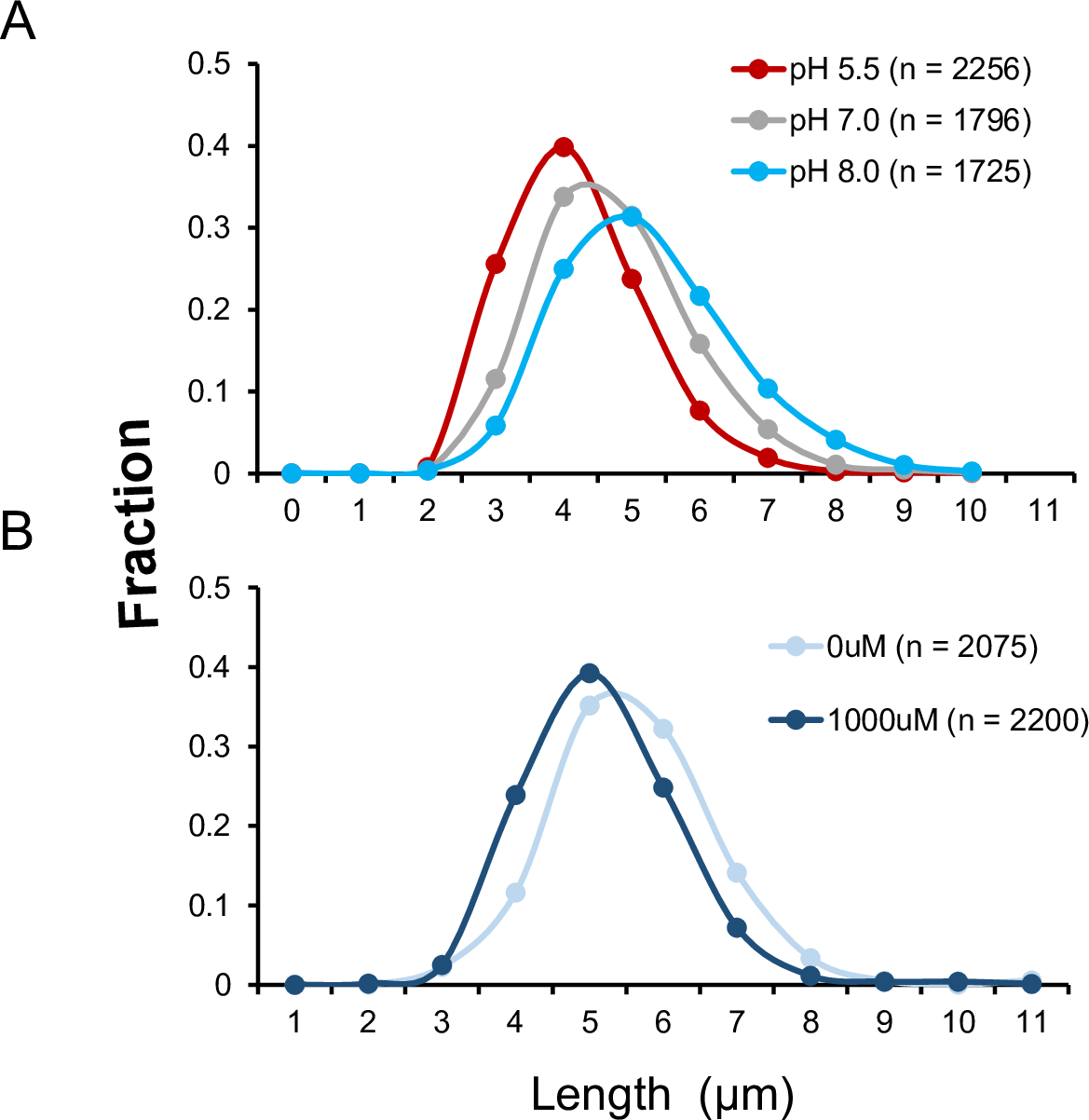

Supplement: S2 Fig — Related to Figs 1 and 5. (TIF) [file pgen.1008685.s002.tif]

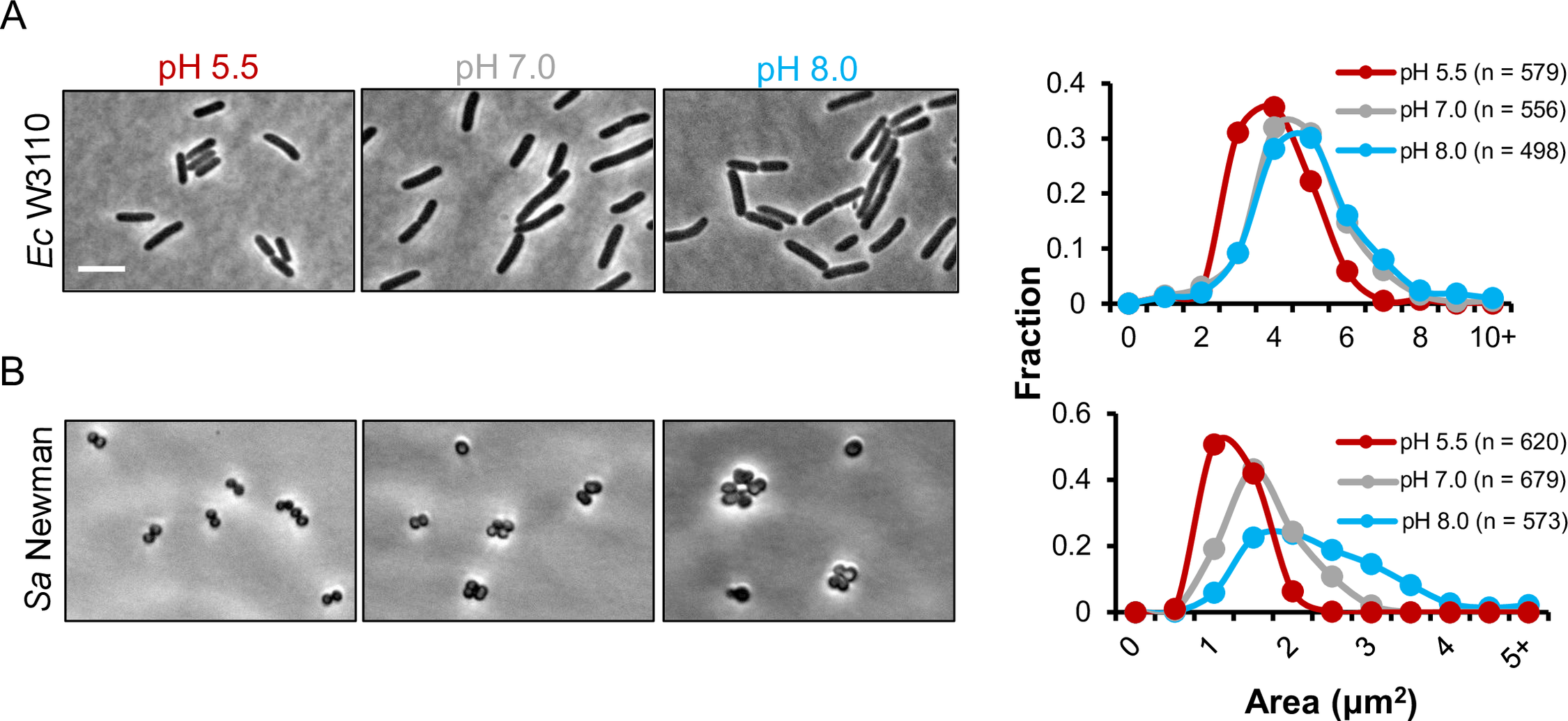

Supplement: S3 Fig — A-B) Representative micrographs and cell area distributions for E. coli strain W3110 grown to steady state in LB + 0.2% glucose (A) and S. aureus strain Newman grown in TSB (B) at pH 5.5, 7.0, and 8.0 and collected for imaging at OD600 ~ 0.1–0.2. Scale bar denotes 5 μm. (TIF) [file pgen.1008685.s003.tif]

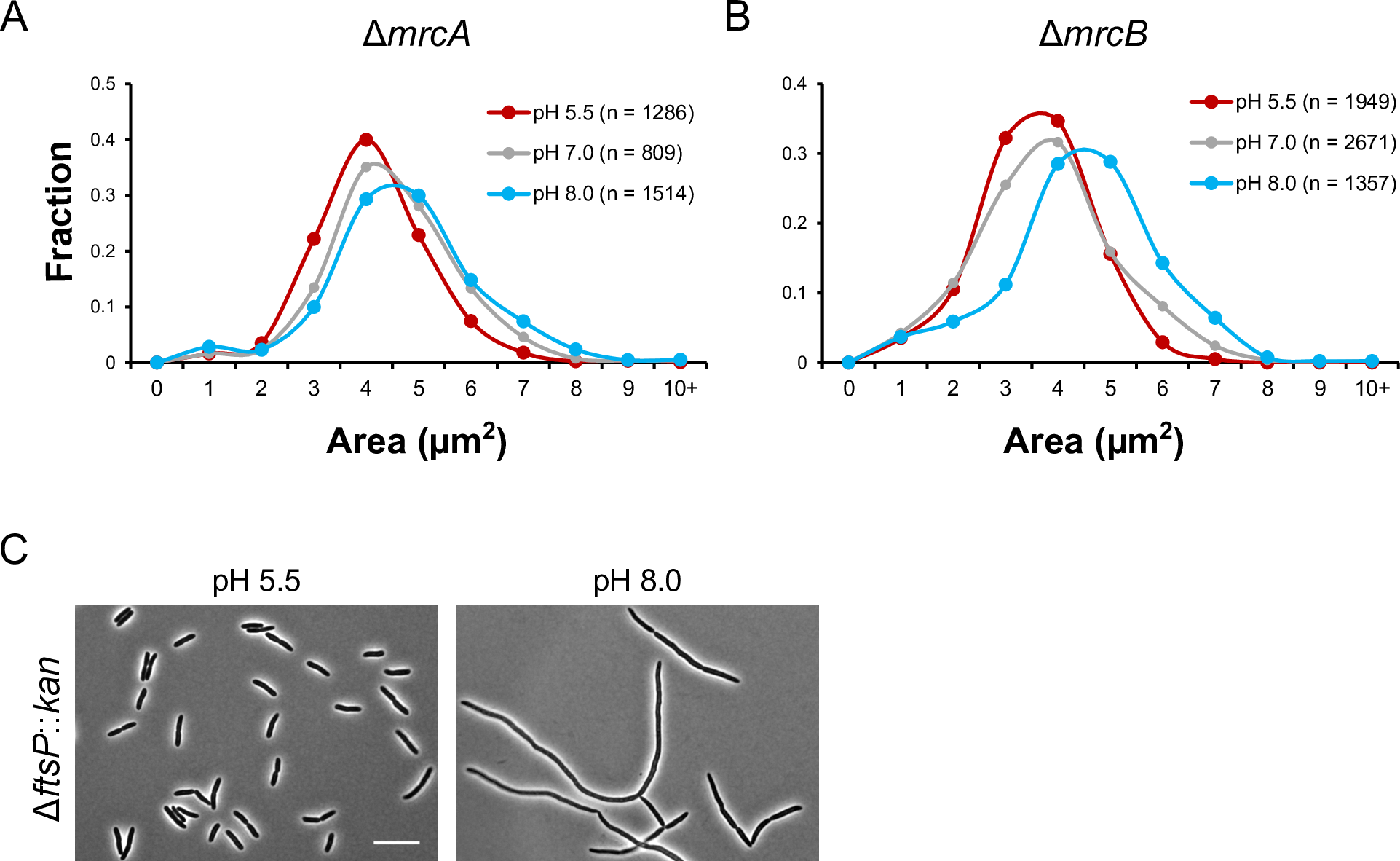

Supplement: S4 Fig — A-B) Cell area distributions for MG1655 strains defective for PBP1a (mrcA::frt, EAM899) and PBP1b (mrcB::frt, EAM696) production during steady state growth in LB + 0.2% glucose at pH 5.5, 7.0, and 8.0. Cells were collected for imaging at OD600 ~ 0.1–0.2. C) Representative micrographs of MG1655 strain defective for FtsP (ftsP::kan, EAM1081) during steady state growth in LB + 0.2% glucose at pH 5.5 (left) and pH 8.0 (right). Cells were collected for imaging at OD600 ~ 0.1–0.2. (TIF) [file pgen.1008685.s004.tif]

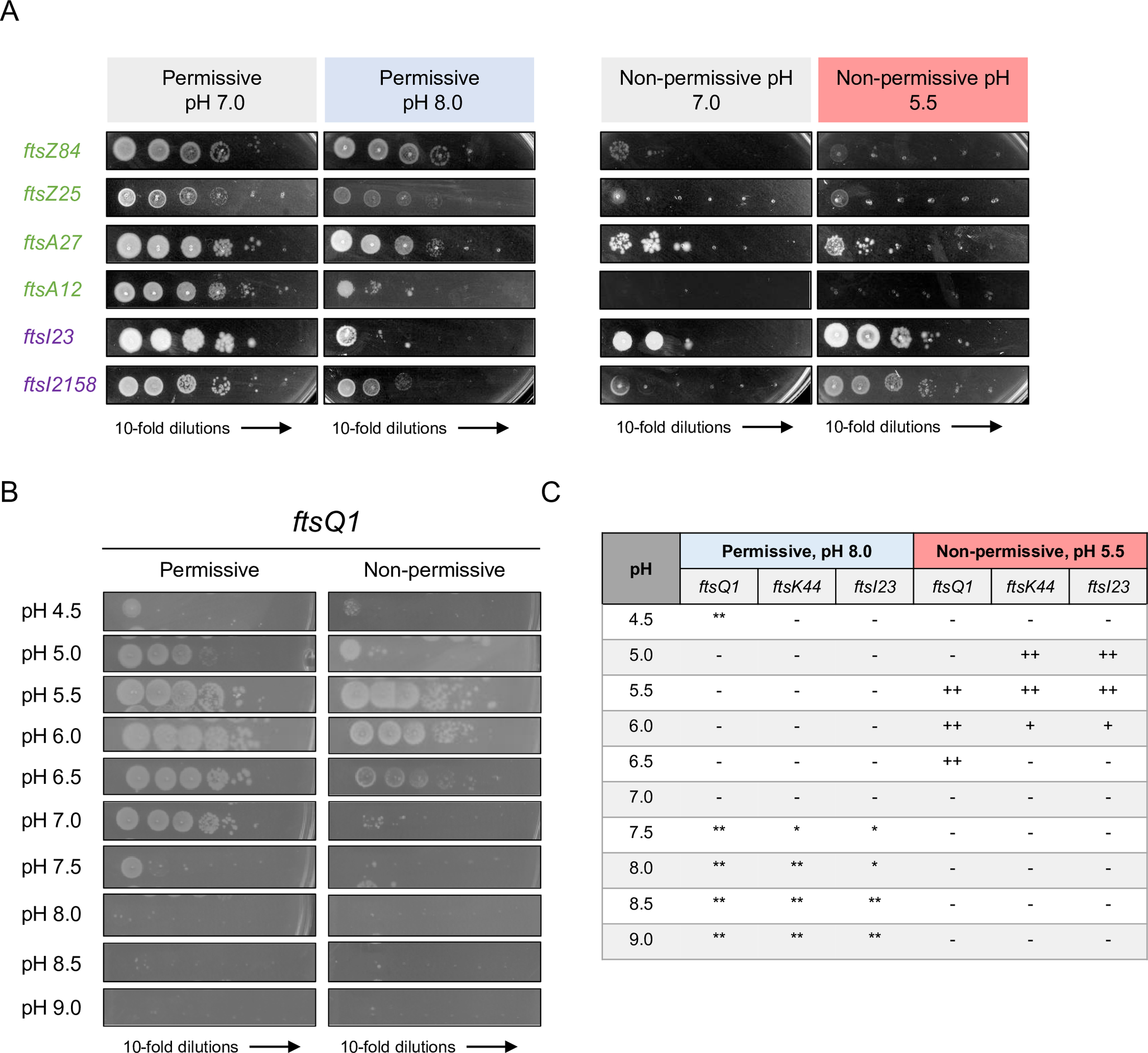

Supplement: S5 Fig — A) Representative plating efficiency for cells producing unique heat-sensitive variants of FtsZ (PAL2452, PAM161), FtsA (WM4107, MM61), and FtsI (WM4649, AX655) during growth at permissive (left) or non-permissive (right) conditions. B) Representative plating efficiency for cells harboring the ftsQ1 allele (EC433) upon exposure to a wide pH range under permissive (left) and non-permissive (right) conditions. C) Table summarizing suppression and enhancement data for strains harboring temperature sensitive variants in late division proteins (EC433, ftsQ1; WM2101, ftsK44; WM4649, ftsI23) across a range of pH conditions. ++, +, and −denote complete, partial, or no suppression at the indicated pH. **, *, and −denote complete, partial, or no enhancement at the indicated pH. (TIF) [file pgen.1008685.s005.tif]

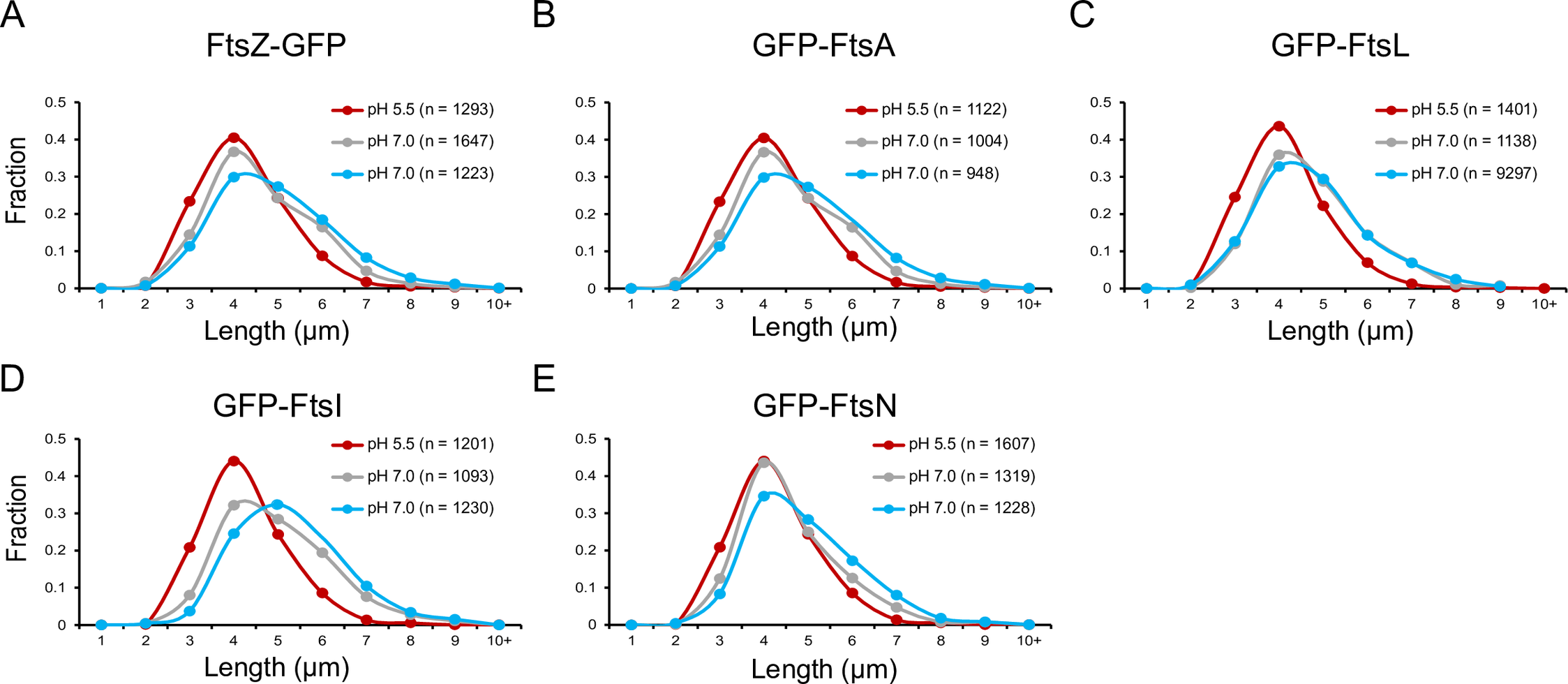

Supplement: S6 Fig — A-E) Cell length distributions of cells overexpressing tagged division proteins, including FtsZ-GFP (A, BH330), GFP-FtsA (B, EAM410), GFP-FtsL (C, PAL3700), GFP-FtsI (D, EAM412), and FtsN (E, EAM621) during steady state growth in LB medium at pH 5.5, 7.0, and 8.0. Cells were collected for imaging at OD600 ~ 0.1–0.2. (TIF) [file pgen.1008685.s006.tif]

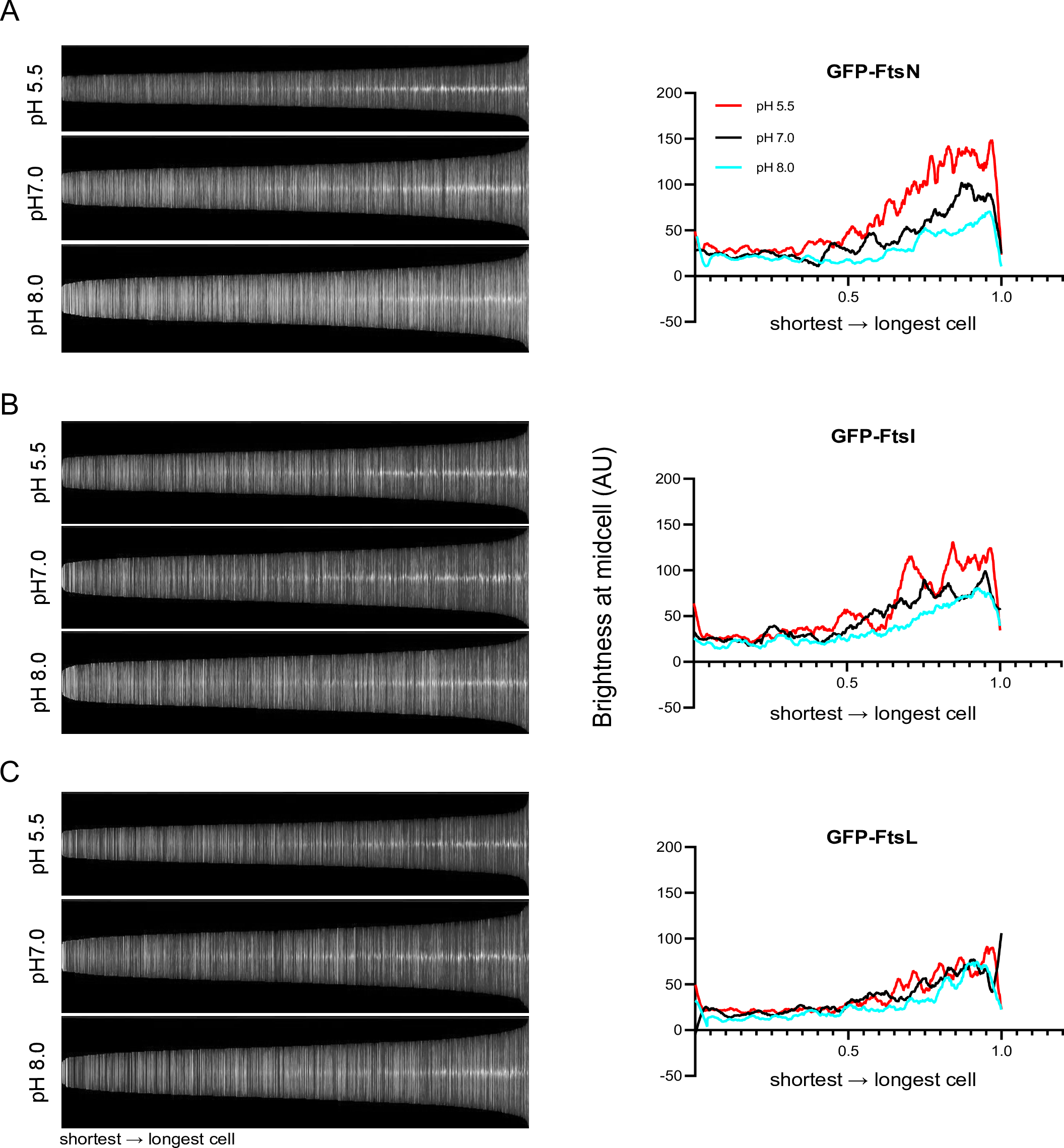

Supplement: S7 Fig — A-C) Mid-cell intensity quantifications (right) and demographs (left) for cells producing GFP-FtsN (A, EAM621), GFP-FtsI (B, EAM412), or GFP-FtsL (C, PAL3700). (TIF) [file pgen.1008685.s007.tif]

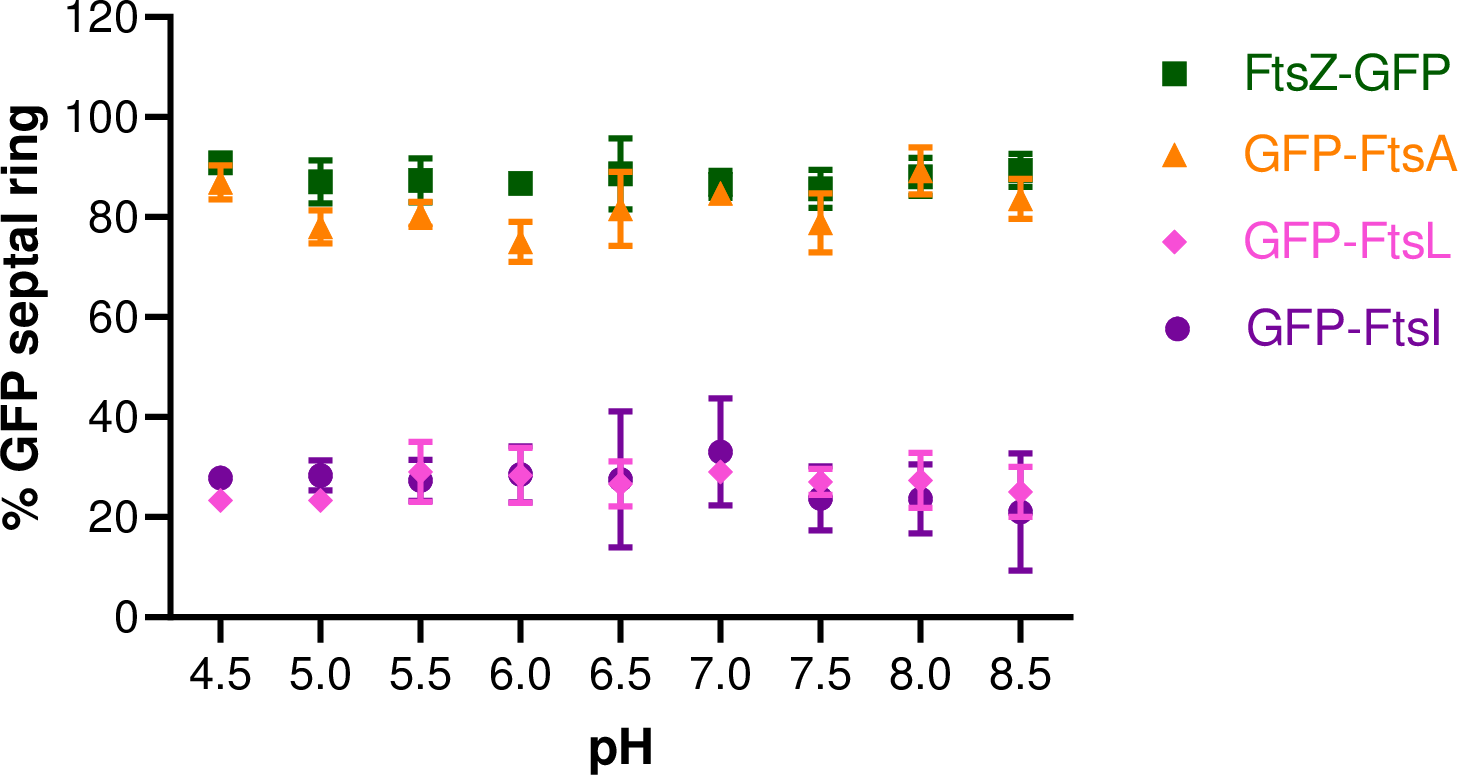

Supplement: S8 Fig — (TIF) [file pgen.1008685.s008.tif]

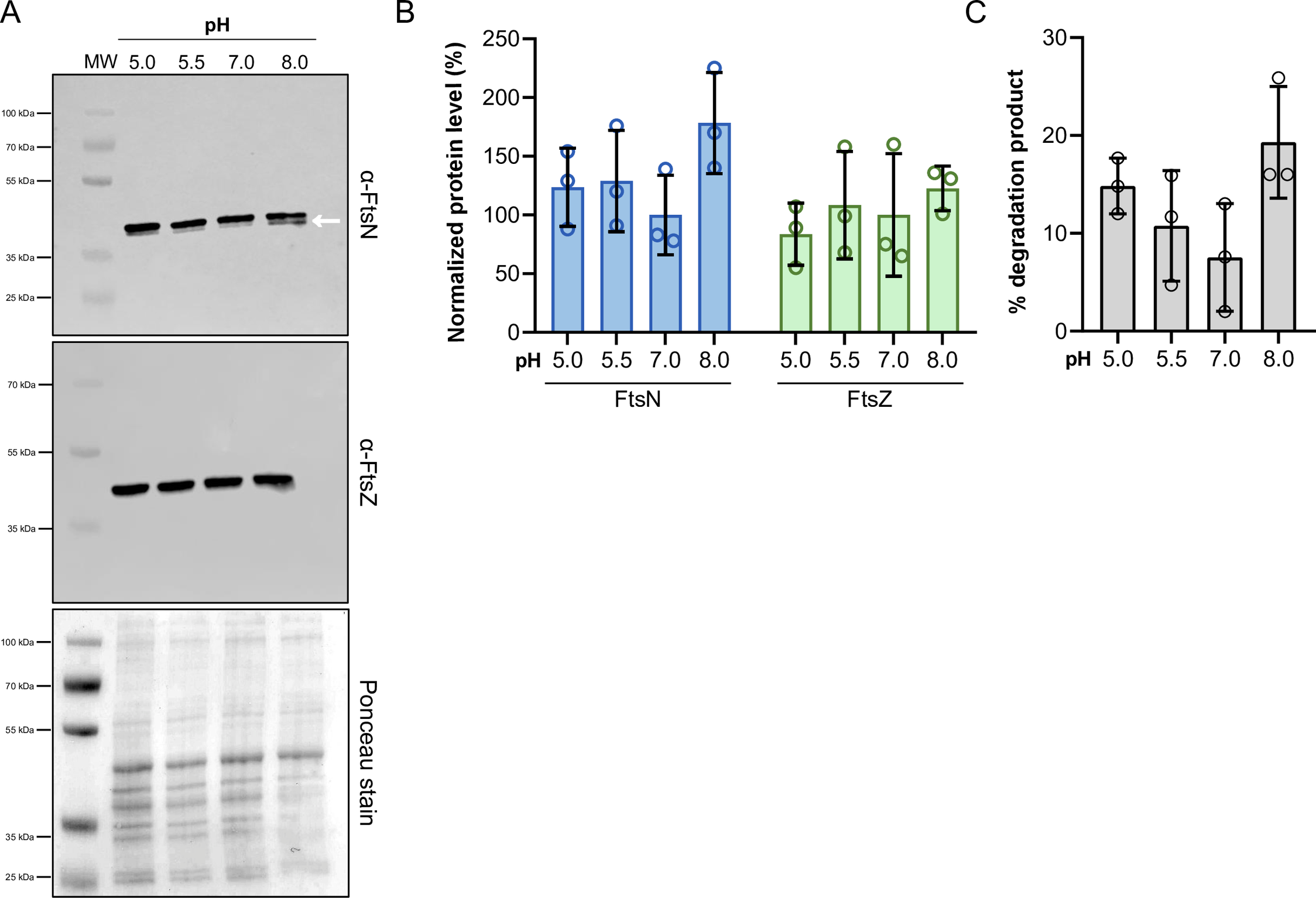

Supplement: S9 Fig — A) Uncropped membrane shown in Fig 3 probed with anti-MBP-FtsN sera (top) and anti-FtsZ sera (middle) or strained with Ponceau reagent for total protein levels (bottom). Arrow indicates degradation or processed FtsN band. B) Quantification of relative FtsN and FtsZ levels as a function of pH. Bars depict mean relative levels of each protein ± SD relative to pH 7.0 from three independent cultures and normalized for total protein load as determined by Ponceau stain. C) FtsN degradation product as a percentage of total FtsN across pH conditions. (TIF) [file pgen.1008685.s009.tif]

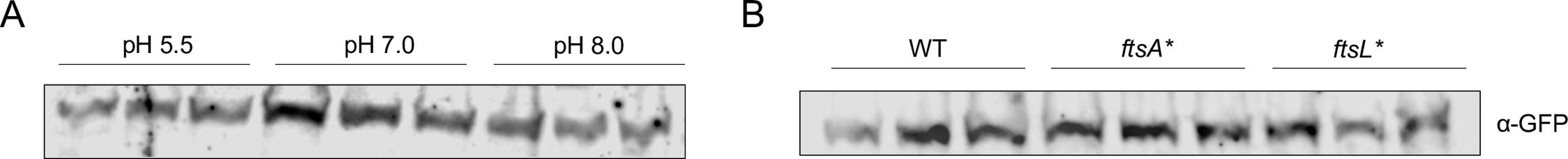

Supplement: S10 Fig — A) Western blot for GFP-FtsN levels (EAM621) from cells grown to steady state in LB medium at pH 5.5, 7.0, and 8.0. Three replicates for each pH condition are shown. B) Western blot depicting GFP-FtsN levels from MG1655 (EAM621), ftsA* (EAM747), and ftsL* (EAM749) grown to steady state in LB medium (pH 7.0). Three biological replicates are shown for each strain. (TIF) [file pgen.1008685.s010.tif]

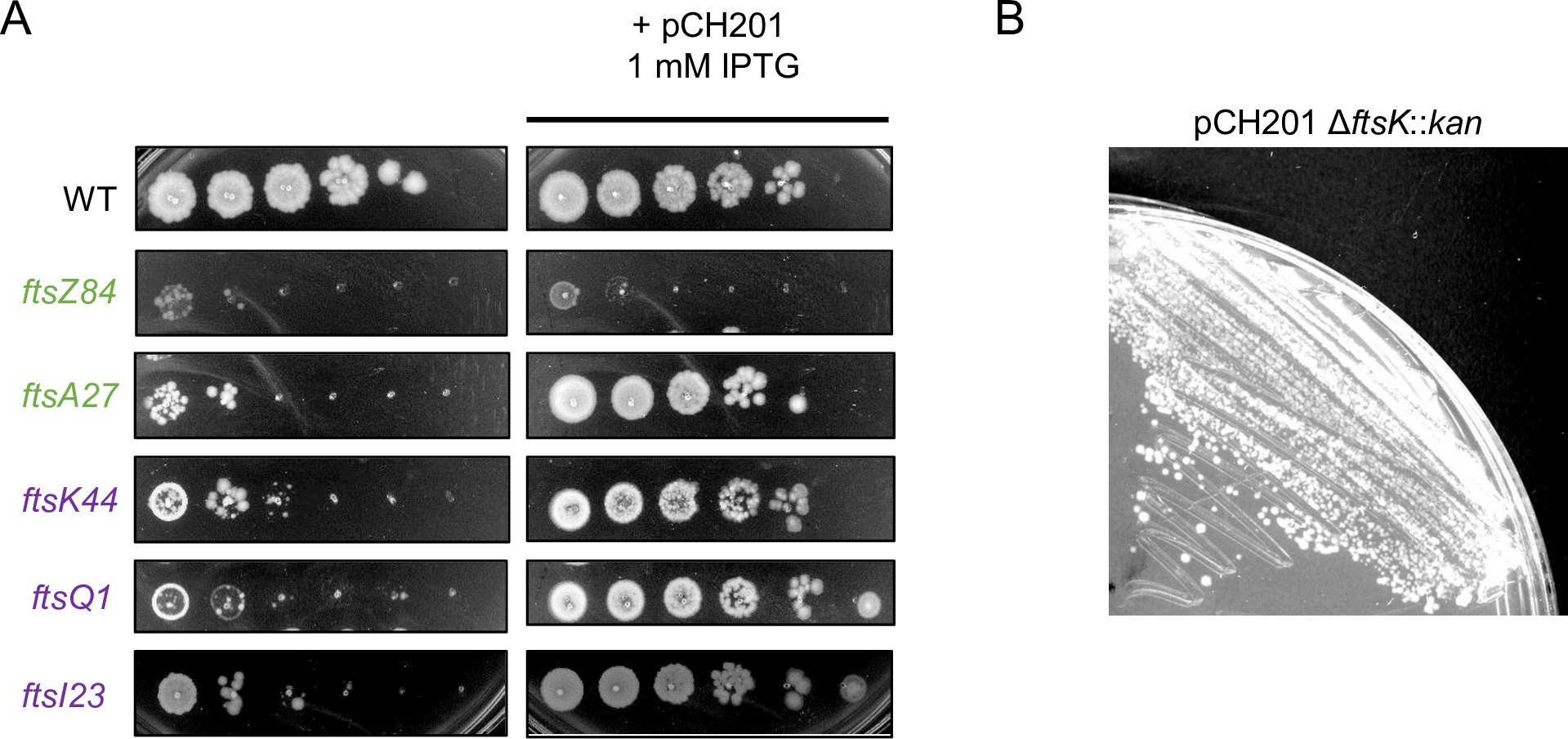

Supplement: S11 Fig — A) Representative plating efficiency for cells producing heat sensitive variants of division proteins (PAL2452, ftsZ84; WM4107, ftsA27; WM2101, ftsK44; EC433, ftsQ1; WM4649, ftsI23) under non-permissive growth conditions in the presence (right) or absence (left) of ftsN overexpression (pCH201; 1 mM IPTG). B) MG1655 can grow in the absence of FtsK (EAM1311) upon ftsN overexpression (1 mM IPTG). (TIF) [file pgen.1008685.s011.tif]

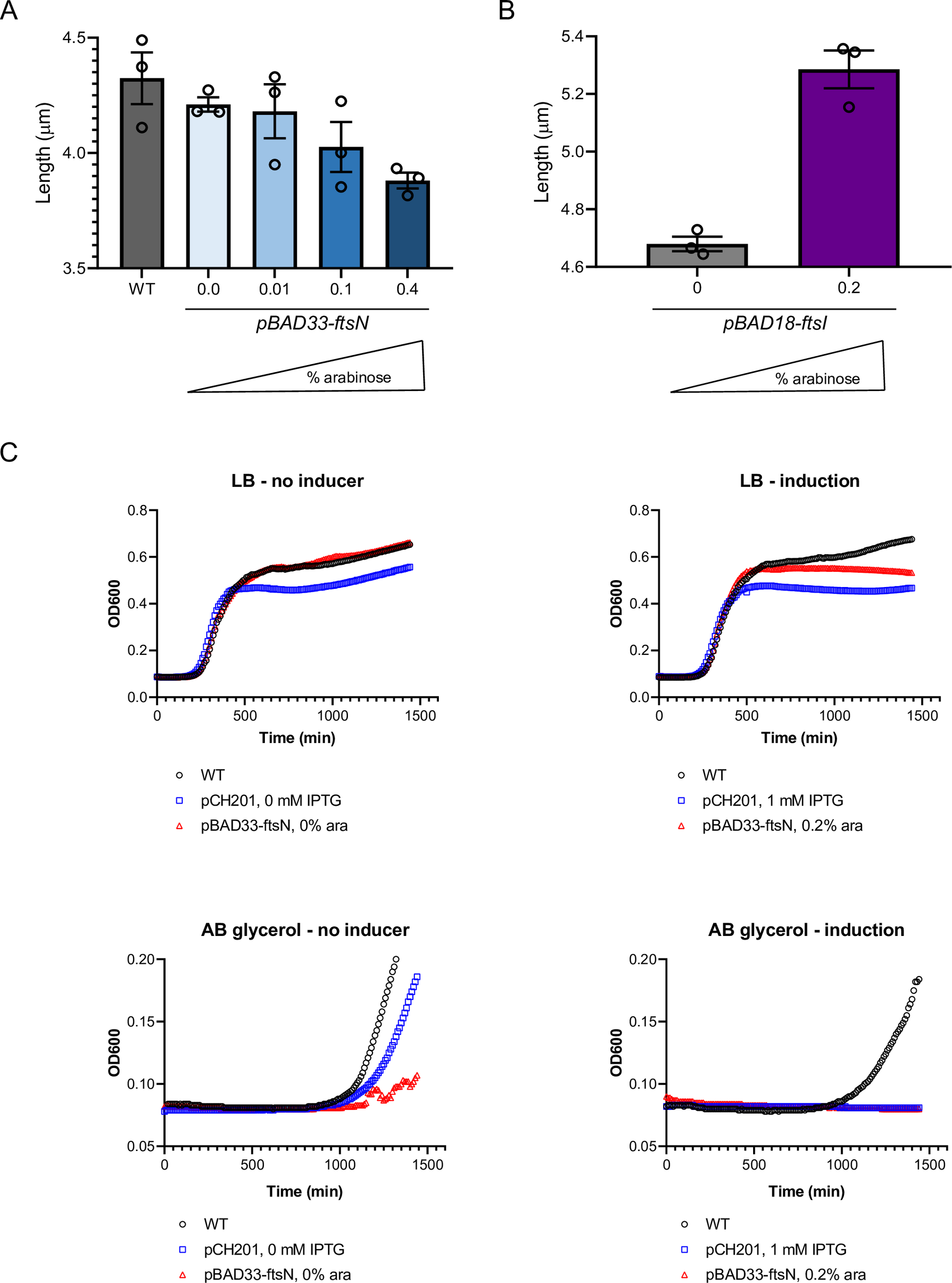

Supplement: S12 Fig — A-B) Cell length of MG1655 producing excess FtsN (pBAD33-ftsN; A) or FtsI (pBAD18-ftsI; B) during steady state growth in LB medium. Cells were collected for imaging at OD600 ~ 0.1–0.2. Bars represent mean cell length ± SEM from three independent biological replicates (n > 200 cells per replicate). C) Representative growth curves for WT (MG1655) cells +/- ftsN overexpression plasmids during growth in LB medium or AB minimal medium + 0.2% glycerol. Cells were grown to steady state in LB medium (uninduced) then inoculated into a 96-well plate in the indicated medium with and without inducer. (TIF) [file pgen.1008685.s012.tif]

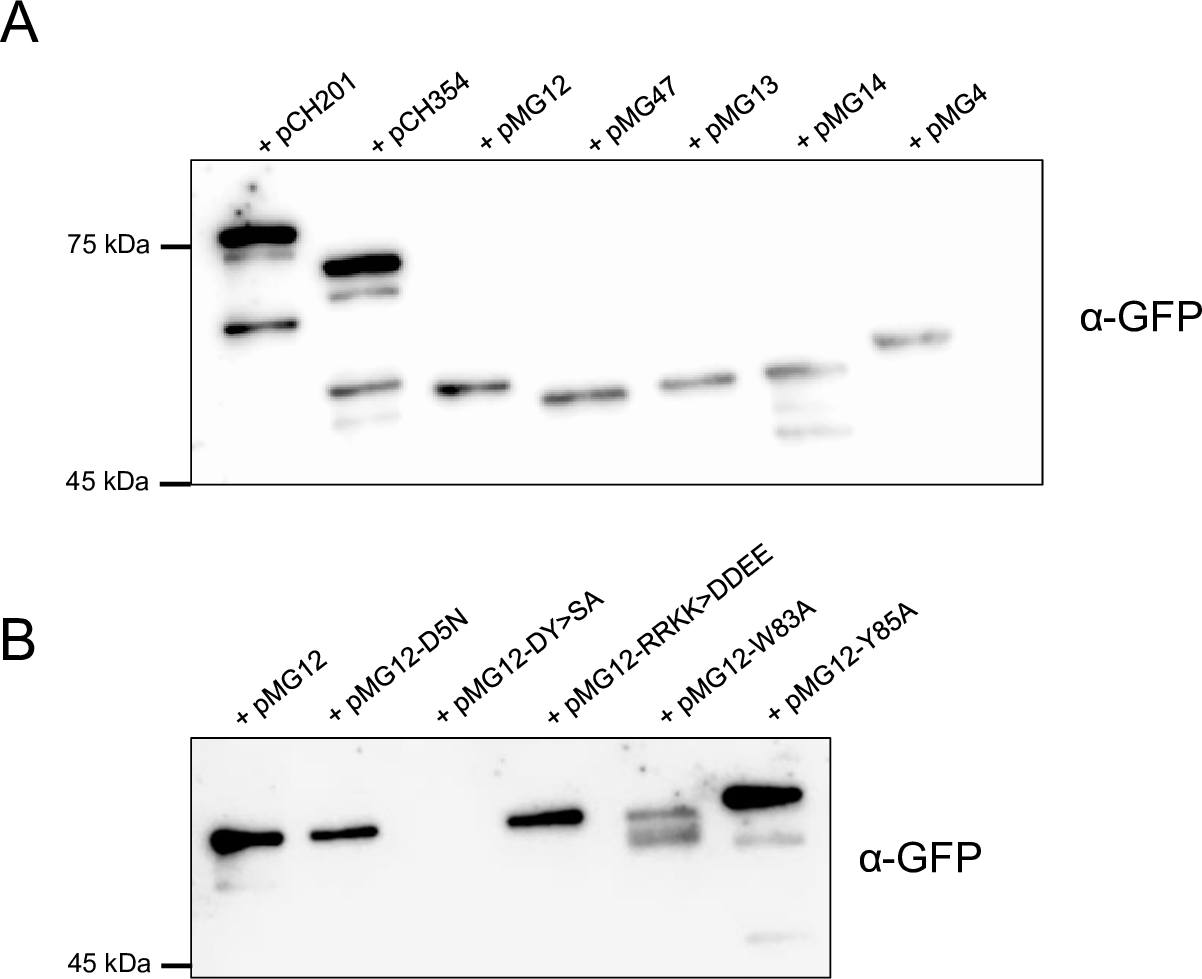

Supplement: S13 Fig — A-B) Representative Western blots for GFP-FtsN truncations (A) or point mutants (B) expressed in MG1655 during steady state growth in LB medium (+1 mM IPTG) and probed with anti-GFP. (TIF) [file pgen.1008685.s013.tif]

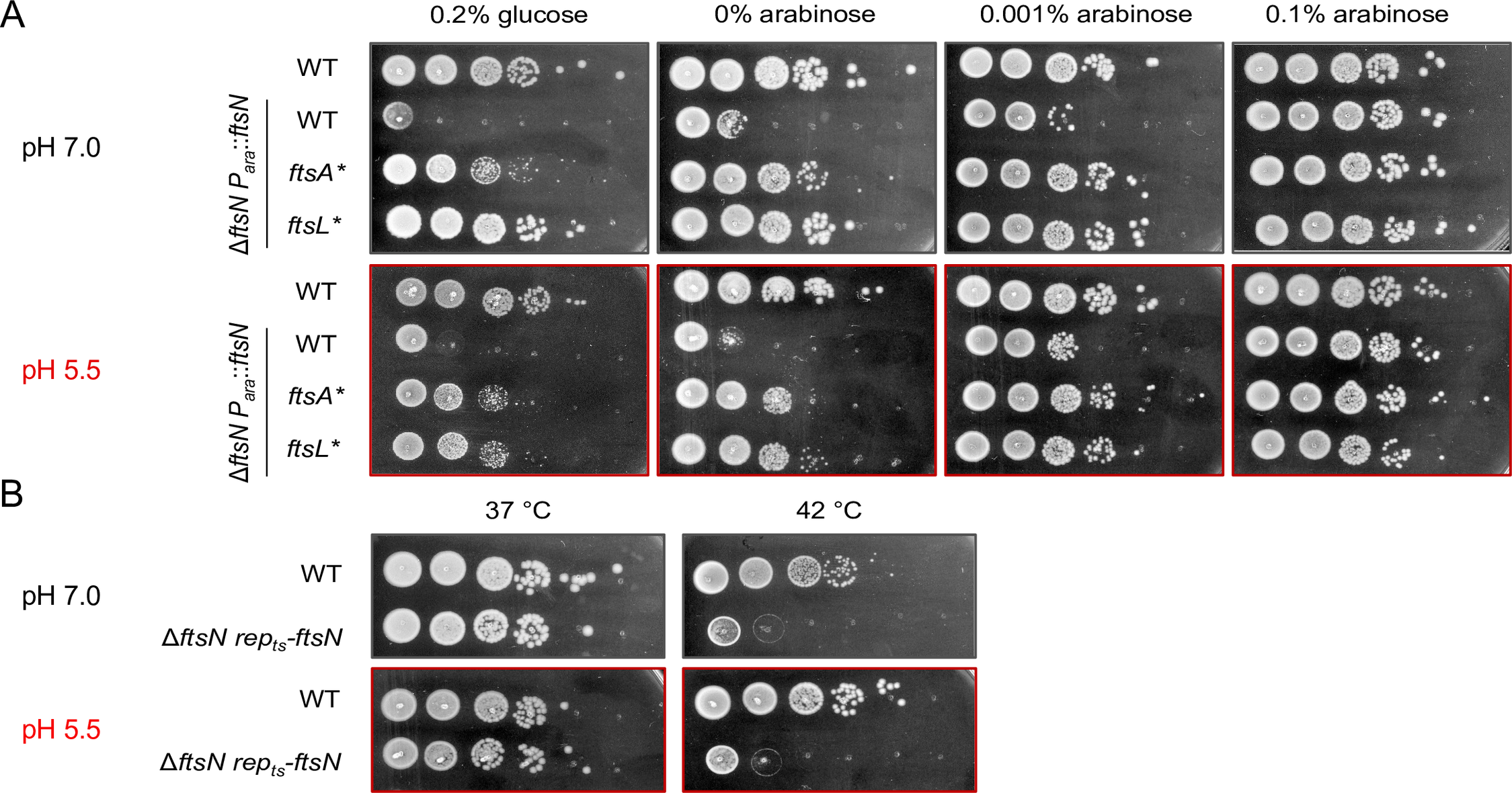

Supplement: S14 Fig — A) Representative plating efficiency for ftsN depletion in WT (HSC074/pBAD33-ftsN), ftsA* (EAM719/pBAD33-ftsN), and ftsL* (EAM723/pBAD33-ftsN) cells at pH 5.5 (bottom) or neutral pH (right) across induction conditions. Image is representative of three biological replicates. B) Representative plating efficiency for temperature-dependent ftsN depletion in WT (MG1655/ Psyn135::ftsN) at pH 5.5 (bottom) or neutral pH (right). Image is representative of three biological replicates. (TIF) [file pgen.1008685.s014.tif]
