# Supplementary material for: pH-dependent activation of cytokinesis modulates Escherichia coli cell size
Source: PLoS Genet. 2020 Mar 23;16(3):e1008685. doi: 10.1371/journal.pgen.1008685 (PMC7117782; doi:10.1371/journal.pgen.1008685)
Supplement: S1 Table — (PDF) [file pgen.1008685.s015.pdf]

Table S1. Bacterial strains and plasmids used in this study.

STRAINS

**Designation**	**Genotype**	**Source[a]**
MG1655 (*E. coli*)	*rph1 ilvG rfb-50 λ- F-*	[3]
W3110 (*E. coli*)	*rph1 IN(rrnD-rrnE)1 λ- F-*	[4]
MC4100 (*E. coli*)	*araD129 ΔlacU169 relA1 rpsL150 thi mot flb5301 deoC7 rbsR F-*	[5]
Newman (*S. aureus)*		[6]
TB28	MG1655 *lacIZYA*::*frt*	[7]
EAM696	MG1655 *mrcB*::*frt*	[8]
EAM899	MG1655 *mrcA*::*frt*	[8]
EAM1081	MG1655 *ftsP*::*kan*	P1(JW2985[b]) x MG1655
CW142	MG1655 *malE*::*kan*	P1(JW3994[b]) x MG1655
BH330	MG1655 P_{lac}-*gfp-ftsZ*	[9]
EC479	MC4100 P_{210}-*gfp-ftsA leu*::Tn*10*	[10]
EAM410	MG1655 P_{210}-*gfp-ftsA leu*::Tn*10*	P1(EC479) x MG1655
PAL3700	TB28 P_{lac}-*gfp-ftsL*	[11]
EC454	MC4100 P_{207}-*gfp-ftsI leu*::Tn*10*	[10]
EAM412	MG1655 P_{207}-*gfp-ftsI leu*::Tn*10*	P1(EC454) x MG1655
EAM621	MG1655 P_{204}-*gfp-ftsN*	[12]
PAL2452	MG1655 *leu82*::Tn*10 ftsZ84*	[13]
WM4649	MG1655 *lacU169 leu82*::Tn*10 ftsI23*	[14]
EC433	MG1655 *leu82*::Tn*10 ftsQ1*	[15]
MM61	MG1655 *leu82*::Tn*10 ftsA12*	[15]
WM2101	MG1655 *lacU169 ycaD::*Tn*10 ftsK44*	[16]
WM4107	MG1655 *lacU16 leu-260*::Tn*10 ftsA27*	[17]
PAM161	*ftsZ25*	[18]

AX655	*ftsI2158*	[19]
BH142	MG1655 *leu82*::Tn*10 ftsA**	[20]
MT13	TB28 *leu82*::Tn*10 ftsL**	[11]
EAM747	MG1655 *leu82*::Tn*10 ftsA** P_{204}-*gfp-ftsN*	P1(EAM621) x BH142
EAM749	TB28 *leu82*::Tn*10 ftsL** P_{204}-*gfp-ftsN*	P1(EAM621) x MT13
MT75	TB28 *ftsK*::*kan*	[11]
EAM1311	MG1655 *ftsK*::*kan*	P1(MT75) x MG1655
HSC074	MC4100 *ftsN*::*kan*	[21]
EAM719	MC4100 *ftsN*::*kan leu82*::Tn*10 ftsA**	P1(BH142) x HSC074
EAM723	MC4100 *ftsN*::*kan leu82*::Tn*10 ftsL**	P1(MT13) x HSC074

[a] Strains constructed by P1 transduction are described using the shorthand: P1(donor) x recipient.
[b] Strains sourced from the Coli Genetic Stock Center [22]

PLASMIDS

**Designation**	**Genotype**	**Source**
pCH201	*bla lacI*^{q} P_{lac}::*gfp-FtsN(1-319)*	[23]
pCH354	*bla lacI*^{q} P_{lac}::*gfp-FtsN(1-243)-le*	[23]
pMG12	*bla lacI*^{q} P_{lac}::*gfp-FtsN(1-105)-le*	[23]
pMG47	*bla lacI*^{q} P_{lac}::*gfp-FtsN(1-90)*	[23]
pMG13	*bla lacI*^{q} P_{lac}::*gfp-FtsN(1-81)-le*	[23]
pMG14	*bla lacI*^{q} P_{lac}::^{ss}*torA-gfp-FtsN(71-105)-le*	[23]
pMG4	*bla lacI*^{q} P_{lac}::^{ss}*torA-gfp-FtsN(241-319)-le*	[23]
pMG12-D5N	*bla lacI*^{q} P_{lac}::*gfp-FtsN(1-105)-le(D5N)*	This work
pMG12-RRKK>DDEE	*bla lacI*^{q} P_{lac}::*gfp-FtsN(1-105)-le(RRKK>DDEE)*	This work
pMG12-W83A	*bla lacI*^{q} P_{lac}::*gfp-FtsN(1-105)-le(W83A)*	This work
pMG12-Y85A	*bla lacI*^{q} P_{lac}::*gfp-FtsN(1-105)-le(Y85A)*	This work

pBAD33-ftsN	*pBAD33-ftsN*	[24]
pLMG173	*pBAD18-ftsI*	[25]
pBL154	*aadA repA(ts) Psyn135::ftsN*	[26]

REFERENCES

1. Vischer NOE, Verheul J, Postma M, van den Berg van Saparoea B, Galli E, Natale P, et al. Cell age dependent concentration of Escherichia coli divisome proteins analyzed with ImageJ and ObjectJ. Front Microbiol. Frontiers; 2015;6: 586. doi:10.3389/fmicb.2015.00586

2. Schindelin J, Arganda-Carreras I, Frise E, Kaynig V, Longair M, Pietzsch T, et al. Fiji: an open-source platform for biological-image analysis. Nat Methods. Nature Publishing Group; 2012;9: 676–682. doi:10.1038/nmeth.2019

3. Guyer MS, Reed RR, Steitz JA, Low KB. Identification of a Sex-factor-affinity Site in E. coli as γδ. Cold Spring Harb Symp Quant Biol. Cold Spring Harbor Laboratory Press; 1981;45: 135–140. doi:10.1101/SQB.1981.045.01.022

4. Bachmann BJ. Pedigrees of some mutant strains of Escherichia coli K-12. Bacteriol Rev. American Society for Microbiology (ASM); 1972;36: 525–557.

5. Casadaban MJ. Transposition and fusion of the lac genes to selected promoters in Escherichia coli using bacteriophage lambda and Mu. J Mol Biol. 1976;104: 541–555. doi:10.1016/0022-2836(76)90119-4

6. DUTHIE ES, LORENZ LL. Staphylococcal coagulase; mode of action and antigenicity. J Gen Microbiol. Microbiology Society; 1952;6: 95–107. doi:10.1099/00221287-6-1-2-95

7. Bernhardt TG, de Boer PAJ. Screening for synthetic lethal mutants in Escherichia coli and identification of EnvC (YibP) as a periplasmic septal ring factor with murein hydrolase activity. Mol Microbiol. John Wiley & Sons, Ltd (10.1111); 2004;52: 1255–1269. doi:10.1111/j.1365-2958.2004.04063.x

8. Mueller EA, Egan AJ, Breukink E, Vollmer W, Levin PA. Plasticity of Escherichia coli cell wall metabolism promotes fitness and antibiotic resistance across environmental conditions. eLife. eLife Sciences Publications Limited; 2019;8: 492. doi:10.7554/eLife.40754

9. Hill NS, Buske PJ, Shi Y, Levin PA. A moonlighting enzyme links Escherichia coli cell size with central metabolism. Casadesús J, editor. PLoS Genet. 2013;9: e1003663. doi:10.1371/journal.pgen.1003663

10. Weiss DS, Chen JC, Ghigo JM, Boyd D, Beckwith J. Localization of FtsI (PBP3) to the septal ring requires its membrane anchor, the Z ring, FtsA, FtsQ, and FtsL. J Bacteriol. American Society for Microbiology (ASM); 1999;181: 508–520.

11. Tsang M-J, Bernhardt TG. A role for the FtsQLB complex in cytokinetic ring activation revealed by an ftsL allele that accelerates division. Mol Microbiol. John Wiley & Sons, Ltd (10.1111); 2015;95: 925–944. doi:10.1111/mmi.12905

12. Westfall CS, Levin PA. Comprehensive analysis of central carbon metabolism illuminates connections between nutrient availability, growth rate, and cell morphology in Escherichia coli. Søgaard-Andersen L, editor. PLoS Genet. Public Library of Science; 2018;14: e1007205. doi:10.1371/journal.pgen.1007205

13. Arjes HA, Lai B, Emelue E, Steinbach A, Levin PA. Mutations in the bacterial cell division protein FtsZ highlight the role of GTP binding and longitudinal subunit interactions in assembly and function. BMC Microbiol. 3rd ed. BioMed Central; 2015;15: 209. doi:10.1186/s12866-015-0544-z

14. Schoenemann KM, Krupka M, Rowlett VW, Distelhorst SL, Hu B, Margolin W. Gain-of-function variants of FtsA form diverse oligomeric structures on lipids and enhance FtsZ protofilament bundling. Mol Microbiol. John Wiley & Sons, Ltd (10.1111); 2018;109: 676–693. doi:10.1111/mmi.14069

15. Chen JC, Weiss DS, Ghigo JM, Beckwith J. Septal localization of FtsQ, an essential cell division protein in Escherichia coli. J Bacteriol. American Society for Microbiology (ASM); 1999;181: 521–530.

16. Haeusser DP, Rowlett VW, Margolin W. A mutation in Escherichia coli ftsZ bypasses the requirement for the essential division gene zipA and confers resistance to FtsZ assembly inhibitors by stabilizing protofilament bundling. Mol Microbiol. John Wiley & Sons, Ltd (10.1111); 2015;97: 988–1005. doi:10.1111/mmi.13081

17. Herricks JR, Nguyen D, Margolin W. A thermosensitive defect in the ATP binding pocket of FtsA can be suppressed by allosteric changes in the dimer interface. Mol Microbiol. John Wiley & Sons, Ltd (10.1111); 2014;94: 713–727. doi:10.1111/mmi.12790

18. Donch J, Greenberg J. Genetic analysis of lon mutants of strain K-12 of Escherichia coli. Mol Gen Genet. Springer-Verlag; 1968;103: 105–115. doi:10.1007/bf00427138

19. Fletcher G, Irwin CA, Henson JM, Fillingim C, Malone MM, Walker JR. Identification of the Escherichia coli cell division gene sep and organization of the cell division-cell envelope genes in the sep-mur-ftsA-envA cluster as determined with specialized transducing lambda bacteriophages. J Bacteriol. American Society for Microbiology (ASM); 1978;133: 91–100.

20. Hill NS, Kadoya R, Chattoraj DK, Levin PA. Cell size and the initiation of DNA replication in bacteria. Burkholder WF, editor. PLoS Genet. Public Library of Science; 2012;8: e1002549. doi:10.1371/journal.pgen.1002549

21. Gonzalez MD, Beckwith J. Divisome under construction: distinct domains of the small membrane protein FtsB are necessary for interaction with multiple cell division proteins. J Bacteriol. American Society for Microbiology Journals; 2009;191: 2815–2825. doi:10.1128/JB.01597-08

22. Baba T, Ara T, Hasegawa M, Takai Y, Okumura Y, Baba M, et al. Construction of Escherichia coli K-12 in-frame, single-gene knockout mutants: the Keio collection. Molecular Systems Biology. EMBO Press; 2006;2: 473. doi:10.1038/msb4100050

23. Gerding MA, Liu B, Bendezú FO, Hale CA, Bernhardt TG, de Boer PAJ. Self-enhanced accumulation of FtsN at Division Sites and Roles for Other Proteins with a SPOR domain (DamX, DedD, and RlpA) in Escherichia coli cell constriction. J Bacteriol. American Society for Microbiology Journals; 2009;191: 7383–7401. doi:10.1128/JB.00811-09

24. Chen JC, Beckwith J. FtsQ, FtsL and FtsI require FtsK, but not FtsN, for co-localization with FtsZ during Escherichia coli cell division. Mol Microbiol. John Wiley & Sons, Ltd (10.1111); 2001;42: 395–413. doi:10.1046/j.1365-2958.2001.02640.x

25. Guzman LM, Weiss DS, Beckwith J. Domain-swapping analysis of FtsI, FtsL, and FtsQ, bitopic membrane proteins essential for cell division in Escherichia coli. J Bacteriol. American Society for Microbiology Journals; 1997;179: 5094–5103. doi:10.1128/jb.179.16.5094-5103.1997

26. Liu B, Persons L, Lee L, de Boer PAJ. Roles for both FtsA and the FtsBLQ subcomplex in FtsN-stimulated cell constriction in Escherichia coli. Mol Microbiol. Wiley/Blackwell (10.1111); 2015;95: 945–970. doi:10.1111/mmi.12906
