# Supplementary material for: pH-dependent activation of cytokinesis modulates Escherichia coli cell size
Source: PLoS Genet. 2020 Mar 23;16(3):e1008685. doi: 10.1371/journal.pgen.1008685 (PMC7117782; doi:10.1371/journal.pgen.1008685)
Supplement: S2 Table — (PDF) [file pgen.1008685.s016.pdf]

Table S2. Impact of pH on cell dimensions of MG1655 in LB medium[a]

pH	Area (μm^2)[a,c]	Length (μm)[a,c]	Width (μm)[a,c]	Mass doubling time (min)[b,c]	*n* cells
4.5	2.75 ± 0.07 (****)	3.42 ± 0.12 (****)	0.85 ± 0.01 (*)	32 ± 2 (****)	2600
5.0	2.93 ± 0.07 (***)	3.45 ± 0.04 (****)	0.89 ± 0.01 (ns)	25 ± 1 (***)	2299
5.5	3.25 ± 0.05 (*)	3.72 ± 0.05 (***)	0.92 ± 0.01 (ns)	22 ± 1 (ns)	2256
6.0	3.33 ± 0.02 (ns)	3.92 ± 0.02 (*)	0.89 ± 0.01 (ns)	22 ± 1 (ns)	1588
6.5	3.58 ± 0.14 (ns)	4.07 ± 0.09 (ns)	0.91 ± 0.02 (ns)	21 ± 1 (ns)	1526
7.0	3.75 ± 0.06	4.23 ± 0.04	0.92 ± 0.01	21 ± 1	1796
7.5	3.78 ± 0.15 (ns)	4.34 ± 0.08 (ns)	0.90 ± 0.03 (ns)	21 ± 1 (ns)	1233
8.0	4.25 ± 0.12 (*)	4.73 ± 0.05 (***)	0.93 ± 0.02 (ns)	21 ± 1 (ns)	1725
8.5	4.44 ± 0.19 (**)	4.93 ± 0.08 (****)	0.93 ± 0.03 (ns)	22 ± 1 (ns)	1298

[a] ± SEM
[b] ± SD
[c] Statistical significance compared to pH 7.0 indicated in parenthesis as determined by a one-way ANOVA corrected for multiple comparisons with Dunnett's test.
