# Supplementary material for: pH-dependent activation of cytokinesis modulates Escherichia coli cell size
Source: PLoS Genet. 2020 Mar 23;16(3):e1008685. doi: 10.1371/journal.pgen.1008685 (PMC7117782; doi:10.1371/journal.pgen.1008685)
Supplement: S3 Table — (PDF) [file pgen.1008685.s017.pdf]

Table S3. Septal ring frequencies across pH conditions in LB medium.

Strain/plasmid	[IPTG] (μm)	pH 5.5 ring frequency (%)[a]	pH 7.0 ring frequency (%)[a]	pH 8.0 ring frequency (%)[a]
BH300 (FtsZ-GFP)	1000	87 ± 4	86 ± 2	90 ± 5
EAM410 (GFP-FtsA)	100	81 ± 2	85 ± 3	87 ± 3
PAL3700 (GFP-FtsL)	100	29 ± 6	29 ± 2	27 ± 6
EAM412 (GFP-FtsI)	2.5	27 ± 2	26 ± 7	23 ± 3
EAM621 (GFP-FtsN)	5	31 ± 2	23 ± 2	14 ± 3
EAM747 (*ftsA** GFP-FtsN)	5	47 ± 9	45 ± 4	35 ± 6
EAM749 (*ftsL** GFP-FtsN)	5	43 ± 2	48 ± 2	38 ± 5
MG1655/pCH201	25	39 ± 5	26 ± 4	15 ± 5
MG1655/pCH354	25	20 ± 3	9 ± 4	3 ± 2
MG1655/pMG12	25	21 ± 2	10 ± 2	5 ± 2
MG1655/pMG13	25	0 ± 1	1 ± 1	1 ± 1
MG1655/pMG12-RRKK>DDEE	25	0 ± 0	0 ± 0	0 ± 0

[a] ± SD
