# Supplementary material for: pH-dependent activation of cytokinesis modulates Escherichia coli cell size
Source: PLoS Genet. 2020 Mar 23;16(3):e1008685. doi: 10.1371/journal.pgen.1008685 (PMC7117782; doi:10.1371/journal.pgen.1008685)
Supplement: S4 Table — (PDF) [file pgen.1008685.s018.pdf]

Table S4. Impact of FtsN overexpression on cell size in LB medium[a]

Plasmid	[IPTG] (uM)	Area (μm^2)[b]	Length (µm)[b]	Width (µm)[b]	Mass doubling time (min)[c]	Ring frequency (%)[c,d]	*n* cells
N/A	N/A	3.81 ± 0.11	4.23 ± 0.07	0.89 ± 0.02	22 ± 2	N/A	7226
pCH201 ($FtsN_{1-319}$)	0	3.89 ± 0.10	4.15 ± 0.08	0.93 ± 0.01	23 ± 2	0.7 ± 0.3	2075
pCH201($FtsN_{1-319}$)	10	3.86 ± 0.10	4.08 ± 0.04	0.94 ± 0.02	22 ± 2	6.3 ± 2.0	1851
pCH201($FtsN_{1-319}$)	100	3.63 ± 0.10	3.80 ± 0.05	0.95 ± 0.01	23 ± 3	34.6 ± 4.5	1859
pCH201 ($FtsN_{1-319}$)	1000	3.42 ± 0.11	3.69 ± 0.05	0.91 ± 0.02	23 ± 2	36.6 ± 7.7	8036
pCH354 ($FtsN_{1-243}$)	1000	3.63 ± 0.11	3.91 ± 0.15	0.92 ± 0.02	20 ± 1	9.3 ± 3.9	2285
pMG12 ($FtsN_{1-115}$)	1000	3.51 ± 0.11	3.88 ± 0.07	0.89 ± 0.03	21 ± 2	10.2 ± 1.6	6170
pMG47 ($FtsN_{1-90}$)	1000	3.82 ± 0.15	4.24 ± 0.06	0.89 ± 0.04	20 ± 1	N.D[e]	1864
pMG13 ($FtsN_{1-81}$)	1000	3.98 ± 0.14	4.30 ± 0.11	0.91 ± 0.03	21 ± 1	0.7 ± 0.8	1835
pMG14 ($FtsN_{71-105}$)	1000	4.01 ± 0.26	4.44 ± 0.13	0.89 ± 0.03	21 ± 1	N.D	2057
pMG4 ($FtsN_{243-319}$)	1000	4.18 ± 0.38	4.71 ± 0.10	0.87 ± 0.07	23 ± 3	49.5 ± 7.2	2778
pMG12-D5N	1000	3.71 ± 0.26	4.12 ± 0.18	0.89 ± 0.05	21 ± 2	N.D	3575
pMG12-RRKK>DDEE	1000	4.22 ± 0.02	4.40 ± 0.06	0.94 ± 0.01	21 ± 1	0 ± 0	1066
pMG12-W83A	1000	3.79 ± 0.13	4.27 ± 0.18	0.88 ± 0.06	22 ± 1	N.D	3083
pMG12-Y85A	1000	3.63 ± 0.29	4.16 ± 0.17	0.86 ± 0.05	22 ± 1	N.D	3470

[a] All plasmids were transformed into the parental strain MG1655

[b] ± SEM
[c] ± SD
[d] pH 7.0
[e] N.D, not determined
