# Supplementary material for: pH-dependent activation of cytokinesis modulates Escherichia coli cell size
Source: PLoS Genet. 2020 Mar 23;16(3):e1008685. doi: 10.1371/journal.pgen.1008685 (PMC7117782; doi:10.1371/journal.pgen.1008685)
Supplement: Attachment — Submitted filename: ResponsestoReviews_V3.pdf [file pgen.1008685.s019.pdf]

Comments to the Authors:
Please note here if the review is uploaded as an attachment.

Reviewer #1: In this manuscript, Mueller and colleagues establish and explore mechanistically the relationship between extracellular pH and cell size in Escherichia coli. They find that cell length is correlated with extracellular pH (lower pH = shorter cells, higher pH = longer cells) implying that cell division is responsive to extracellular pH. They use genetics and imaging to implicate the late-arriving division protein FtsN as a potential mediator of this response. FtsN localizes more frequently and robustly to the division site at low pH than at high pH. In addition, overexpression of ftsN or ftsN truncation variants that include the cytoplasmic domain and CCD is sufficient to induce cell shortening at neutral pH. Hyperactivating mutants in ftsA or ftsL previously shown to bypass essentiality of ftsN render cell division insensitive to pH, though FtsN is more robustly recruited to the division site in those mutants. Collectively, their data lead the authors to propose that in E. coli, acidic pH leads to hyper-recruitment of FtsN, which then leads to activation of the division machinery. They further propose positive feedback from division activation to recruitment of FtsN to robustly trigger cell division.

Overall, the manuscript is clearly presented and, for the most part, logical. The conclusion that activation of cell division is responsive to extracellular pH is well-supported and likely to be of broad interest. However, the conclusion that FtsN is the critical mediator of this regulation in E coli is not sufficiently supported by the data presented. My specific comments are as follows:

Major:

1. The implication of FtsN in mediating pH-responsiveness of the division machinery came initially from localization analysis of 5 division proteins (FtsZ, FtsA, FtsL, FtsI, and FtsN) at varying pH. Ideally localization of other late division proteins should be tested, as well (i.e. FtsW and FtsQ), to gain confidence in the specificity of FtsN enrichment at low pH. Although FtsN is canonically thought to be the last recruit to the divisome in E coli, following FtsI, alternative routes to FtsN recruitment have been suggested under some conditions. Moreover, from supplementary figure S7, GFP-FtsI appears moderately enriched at midcell at lower pH, as well. In addition, it would be useful to have the same full set of data/data analysis presented for each protein imaged either in a main figure or supplement (i.e. for FtsZ, there are images shown in Figure 3D but no demograph or fluorescence quantification as in Figure S7 and, conversely, for FtsI no cell images are shown, but demograph and fluorescence quantification is included). Finally, in the images shown in Figure 3D and E, the total fluorescence appears to decrease as pH increases for both FtsZ-GFP and GFP-FtsN. Is that the case?

We have added micrographs for all tested fusion proteins to Figure 3, as well as septal ring frequency measurements across an extended range of pH conditions (pH 4.5-8.5) in Figure S8. Unlike GFP-FtsN, we did not observe a consistent, pH-dependent trend for any of these fusions in septal ring frequency. Although the fluorescence intensity per cell (normalized to cell area) does decrease slightly in alkaline media (see below), only GFP-FtsN septal ring frequency changes as a function of pH (Figure 3, S8). While a valid concern, if this was the cause of the observed change in ring frequency, we would expect all of our fusions to exhibit a similar reduction in ring frequency in alkaline media. This suggests to us that this is not a confounding factor of our analysis.

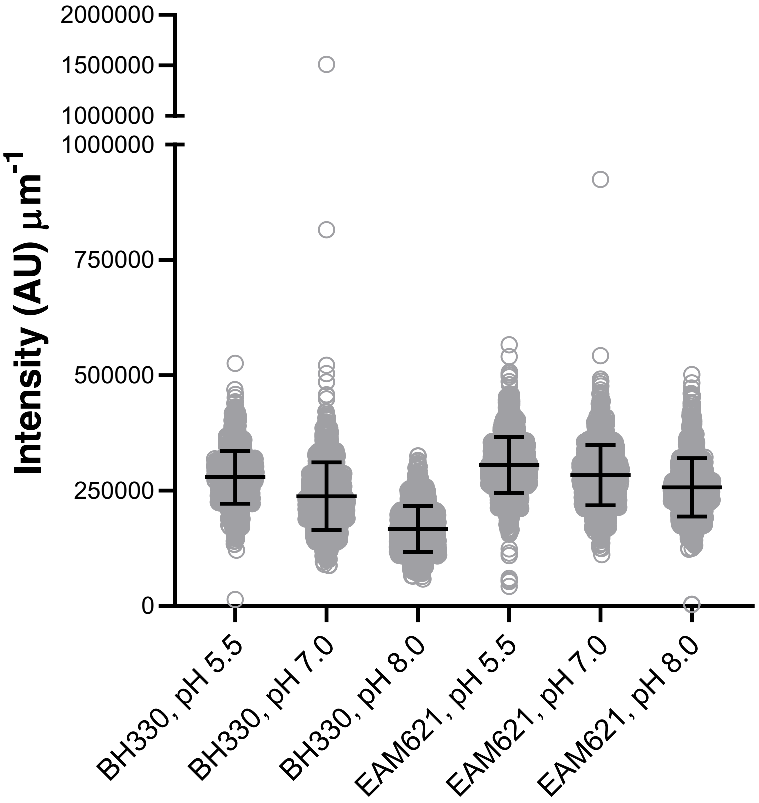

We wish to point out that our aim in comparing the septal ring frequency of the selected fusion proteins was never to conduct an exhaustive examination on the effect of pH on divisome assembly; instead, we sought to narrow down which phase(s) of division were pH responsive to focus our subsequent investigation. Two lines of evidence support FtsN recruitment as pH sensitive: 1) FtsN was the only divisome component to consistently display a positive, pH-dependent change in septal localization frequency and 2) fluorescence intensity quantification confirmed that FtsN is enriched at midcell earlier in the division cycle (i.e. at a reduced cell size) in acidic medium (0.35 point in cell cycle) compared to alkaline medium (0.65 point in cell cycle). Although we agree that there is slight enrichment of GFP-FtsI at midcell in acidic medium, it is relatively modest compared to the change observed in GFP-FtsN intensity, and it does not exhibit a consistent change in septal localization frequency across a wider pH range (Figure S8). This led us to focus our present investigation on FtsN.

While we agree the midcell enrichment of FtsN alone is insufficient evidence to draw the conclusion that FtsN is the pH responsive element of the divisome, we present substantial additional evidence independently supporting a role for FtsN in pH-mediated division activation. 1) Overproduction of FtsN reduces size in rich media (discussed more below). 2) Overproduction of FtsN suppresses the heat sensitivity of a subset of conditional sensitive division mutants similarly suppressed by acidic pH. 3) Growth in alkaline media leads to an increase in cell chaining, a phenotype associated with defects in FtsN (Peters et al., J. Bacteriol., 2011 and others). 4) Unlike *ftsA** and *ftsL**, WT cells cannot tolerate FtsN depletion in acidic media, supporting an role for FtsN in low pH-dependent division activation (discussed more below). Altogether, it is the sum of this evidence that implicates FtsN as an important mediator, although perhaps not the sole determinant of (lines 423-434), pH-dependent division activation.

2. The second piece of evidence implicating FtsN in pH-responsiveness of division is the observation that overexpression of ftsN or ftsN truncations encoding the cytoplasmic region and CCD cause cell shortening at neutral pH. The authors note that two other previous reports of ftsN overexpression in E. coli did not observe cell shortening and actually reported toxicity and cell lengthening. The authors do not discuss why their results might differ from previously published work. Given that this is the only functional evidence presented in the current manuscript directly linking FtsN enrichment to divisome activation, this requires further elaboration. Did the authors try to acquire the constructs used in prior work and test them in their hands? Did they test overexpression of untagged ftsN truncation/mutant variants?

Closer review of the literature indicates that we are not the only ones to observe a decrease in size during *ftsN* overexpression: Modell and Laub (PLOS Biology, 2014) also observed a decrease in size upon overexpression of *gfp-ftsN* in *C. crescentus*. At the same time, one difference between our present work and that of groups reporting FtsN toxicity is media composition. Specifically, several groups find FtsN overproduction is toxic during growth in minimal media (Sekar et al., Mol. Sys. Biol., 2018; Aarsman et al., Mol. Microbiol., 2004). In contrast, we conducted our experiments in rich medium as did Modell and Laub, making it possible that *ftsN* overexpression is more deleterious under slow growth conditions.

To determine if media composition is sufficient to explain the differences in our results, we grew WT cells +/- either tagged or untagged *ftsN* overexpression plasmids (pCH201 [*plac*::*gfp-ftsN*], *pBAD33-ftsN*) to exponential phase in LB media (no inducer), washed the cells, then diluted them into LB media or minimal media (AB + 0.2% glycerol) with and without inducer. As we previously observed, cells overexpressing *ftsN* grew nearly identically to the parental strain in rich media. However, in minimal media, *ftsN* overexpression led to a dramatic growth defect, even in the absence of inducer for one of our constructs (*pBAD33-ftsN*). Representative growth curves are now shown in Figure S12, and the results are discussed in lines 297-301.

Why *ftsN* overexpression is deleterious under nutrient poor conditions is not clear. The requirement to maintain division protein stoichiometry may be more stringent during slow growth and/or a reduction in progression through the cell cycle may leave *E. coli* more sensitive to premature activation of constriction. Deconvoluting the contribution of nutrient derived growth rate and strain background to FtsN toxicity is beyond the scope of this study but an interesting problem for future work.

3. The authors demonstrate that E. coli bearing hyperactivating mutants of ftsA and ftsL that bypass essentiality of ftsN do not change their length in response to pH. In those strains, GFP-FtsN is hyper-enriched at midcell. This led the authors to suggest positive feedback from division activation to FtsN recruitment, which makes sense. However, this brings the chicken-or-egg problem to the pH responsiveness, as the authors note on page 12. Is GFP-FtsN enriched at midcell at low pH, leading to division activation as the authors propose? Or is the divisome hyperactivated at low pH through an FtsN-independent mechanism, causing enrichment of GFP-FtsN at midcell through positive feedback? The authors try to test this by determining if lower levels of FtsN might be sufficient for growth at low pH, as they are in ftsA* and ftsL* mutants, and find that they are not (at least at the arabinose concentration tested - might testing intermediate concentrations be informative?). This suggests that low pH does not hyperactivate division in the same way the ftsA or ftsL mutants that bypass ftsN do, but given the lack of

mechanistic detail of the activation pathway for division, a negative result here is insufficient to rule out the possibility that division is hyper activated at low pH through a mechanism independent of FtsN enrichment. Given the broad conservation of pH-responsiveness of cell division, but lack of broad conservation of FtsN, an FtsN-independent mechanism of pH responsiveness seems likely (perhaps working in concert with low pH-mediated FtsN enrichment in E. coli).

We agree with the reviewer that our data cannot definitively pinpoint FtsN as the sole driver of pH-dependent changes in cell size and cell division. It is challenging to do this, as FtsN is essential and its precise role in cell division is poorly understood. In light of this, we have significantly revised our conclusions to emphasize that while we have substantial data to support a role for FtsN in these processes, we cannot exclude the possibility that other components of the division machinery are also pH responsive.

We respectfully disagree with idea that conservation of pH-responsiveness of cell division among diverse bacterial species suggests a conserved mechanism. Although carbon availability affects the size of many bacterial species, the mechanistic underpinnings of this phenotype are multifactorial and significantly differ between *E. coli* and *B. subtilis*. Like nutrient availability, pH affects numerous processes within the cells, including but not limited to protein-protein interactions, protein stability and/or activity, the proton motive force, and cellular metabolism. Thus, we anticipate the mechanisms underlying pH-dependent changes in size also be multifaceted and is likely to differ between species (see lines 423-434).

At the request of the reviewer, we have also performed experiments to test the effect of pH on FtsN depletion at intermediate concentrations of arabinose, as well as in a separate depletion construct (pBL154 [*aadA repA(ts) Psyn135::ftsN*]). In each of these cases, the extent FtsN depletion tolerated is independent of pH (Figure S14). This reinforces our conclusion that low pH-mediated division activation requires FtsN in *E. coli*.

4. A number of conclusions are overstated:
a. lines 34-36: "…environmental pH impacts the length at which cells divide by altering the ability of the terminal cell division protein FtsN to localize to the cytokinetic machinery and activate division." The localization is fairly well-supported, but there is no evidence presented that supports the conclusion that pH impacts the ability of FtsN to activate division. Do the authors mean "to localize to the cytokinetic machinery where it activates division"?

We have rewritten this sentence as the reviewer suggested.

b. lines 282 and 927: "Acidic pH activates division through FtsN". This section and figure title are overstated. The authors show that ftsA and ftsL hypermorphs are not responsive to pH, that FtsN is enriched at midcell in those backgrounds, and that FtsN is not dispensable at low pH. These data are not sufficient to draw the conclusion that low pH activates division through FtsN.

We have rewritten the section headings to be more conservative.

c. line 310: "…our data indicate FtsN is necessary and sufficient for low pH-mediated division activation…". The overexpression suggests that FtsN is sufficient to induce division hyper-activation, but as that experiment was performed at neutral pH it does not show that FtsN is sufficient to activate division at low pH. Necessity would derive from non-responsiveness of cells

lacking FtsN to pH, but as ftsN is apparently essential for growth at all pHs, this cannot be directly assessed.

This sentence has been eliminated in the revised manuscript.

Other specific points:

5. line 153: “In the final phase of division in proteobacteria, FtsN accumulates at mid-cell and is believed to “trigger” septal PG synthesis…” This has only been shown in E. coli. Though FtsN is conserved in proteobacteria, in Caulobacter it is recruited well before FtsW or FtsI. I know of no evidence in organisms outside of E. coli that implicates FtsN as a trigger for division.

Corrected. We appreciate the reviewer bringing this to our attention.

6. line 217: “Because populations of E. coli cell…” should be “E. coli cells”

Corrected.

7. line 248-9. Which strain in Table S4 is this section referring to? Was this supposed to be Table S5?

It refers to EAM621 (GFP-FtsN) in Table S4.

8. Related to point 7 - it would be really helpful to have brief strain descriptions in each of the tables (e.g. Table S4, S5) to allow the reader to quickly determine what is being expressed in each case. In addition, having relevant strain numbers listed in the figure legends to allow the reader to determine what strain is being used in each experiment would be helpful.

Agreed. We have added brief strain descriptions, as well as updated figure legends with appropriate strain and plasmid identifiers.

9. line 313: “… influence in its septal…” should be “influence on”

Corrected.

10. lines 337-338: It seems relevant to cite reference 20 here, as they demonstrated an effect of division hypermorphs on cell size homeostasis.

We have rephrased this sentence to clarify that our focus is on environmentally determined changes in cell size homeostasis.

11. Figure 5 legend - there is no legend for 5C, and the legend for 5D is mislabeled as C. At what pH was GFP-FtsN localization assessed in 5C?

Corrected.

12. Figure 6. Label on the Y-axis of the graph in A should be “septal” not “septa”

Corrected.

13. Supplemental tables S1 and S2. Arranging strains and plasmids alphabetically by name would help with ease of reference.

We debated the value of either alphabetical and experiment-based/chronological arrangements of the strain and plasmid tables, and ultimately decided the latter would be more useful.

14. Tables S3 and S5. Please define “MDT” in the legend.

Amended to “mass doubling time.”

Reviewer #2: This well written manuscript of Mueller et al. describes the effect of medium pH on cell division and pin-point the activity to FtsN in an intelligent stepwise genetic dissection approach. The importance resides in the fact that they show that cell size is not simply determined by growth rate, and that several cell division mutants can be bypassed or enhanced by changing the pH. This will facilitate the research in cell division since it opens new avenues to create conditional lethal mutations, a key tool in the investigation of bacterial cell division.

The results are clearly presented and build up logically, except for the last two figures, which might be more logical to reverse. Also, I do not necessarily agree with some of the sub-conclusion they pose (see details below). One additional result that I completely cannot understand is how the SPOR domain by itself (GFP-FtsN(243-319)) can localize at the cell division site (Figure 6A) since this domain would than need an N-terminal signal peptide to get transported into the periplasm. Maybe I missed something, but the authors have to make clear how this is possible.

Detailed comments:
l.59: , size, is

Sentence has been rephrased for clarity.

l.71: added

Sentence has been rephrased for clarity.

l.83-86: it would be reasonable to add the effect of pyruvate kinase in B. subtilis (Monahan, 2014, MBio).

Citation added.

l.95: as divisome

Corrected.

l.122: size iso area?

Size refers to cell area here.

Indicate in S3 what MDT is

Corrected in figure legend. MDT refers to “mass doubling time”

l.135: maybe mention why W3110 was chosen?

Added.

l.225: This conclusion is not correct since the localization of FtsA increases slightly but significantly in Fig. 3B

Sentence has been rephrased for clarity.

l.232: in Figure S7 there is also a slight enhancement in FtsI?

In light of this comment, we went back and examined GFP-FtsI septal ring frequency across a wider pH range (pH 4.5-8.5). Unlike GFP-FtsN, we did not observe a consistent, pH-dependent trend in GFP-FtsI septal ring frequency, reinforcing our model that changes in FtsN localization are the primary mechanism underlying pH dependent changes in cell size.

l.244: you cannot enhance an interaction by overexpression (you can increase the chance of interaction).

Sentence has been rephrased for clarity.

Since Figure 4 discusses the truncation study, the question immediately arises how these truncations behave at different pHs. Therefore it might make more sense to follow with the results that are now presented in Figure 6A. This might make the reasoning also easier (see comments below).

We agree with the reviewer and have rearranged the order of the figures for optimal flow. The localization of the truncation mutants (previously Figure 6) is now Figure 4, followed by assessment of the FtsN truncations on cell size (previously Figure 4), and then analysis of the hypermorphic division mutants (previously Figure 5).

l.286 This reasoning is confusing. Firstly, the explanation stated here is not in line with the two models listed on page 10 line 234-236. Secondly, the suggested activation of FtsA by FtsN has not been mentioned before. Only that FtsN interacts with FtsA. The activation, which is not the same as binding, of FtsQLB, has also not been mentioned earlier on. In any case, because it is not known how those hypermorphic FtsA and FtsL mutants bypasses the need for FtsN it is very hard to draw clear conclusions from negative results, i.e. no effect of pH in these mutants.

As suggested by reviewer 1, we have significantly revised our conclusions in this section.

We have also added lines to the discussion (lines 291-400) to highlight recent work that illuminates the mechanistic underpinnings of FtsN-mediated “activation” of FtsA and the septal cell wall synthesis machinery (see Baranova et al. Nature Microbiol., 2020 and Yang et al., BioRxiv, 2019.

l.298: The model that pH regulates FtsA does not make sense since it is dismissed on forehand as it is a cytoplasmic protein, as is also stated in line 323.

We have eliminated line 323 from the text.

l.316: How does GFP-FtsN241-319 gets out of the cell without a signal sequence?

This truncation has an N-terminal TorA signal sequence, targeting it to the periplasm through the twin arginine translocon (genotype: P_{lac}::*sstorA-gfp-FtsN*[241-319]). The text has been updated to emphasize this (lines 307-310)

In the Discussion it would be good to mention that FtsN is not present in Staphylococci, so that other late proteins must be affected in this organism.

This is mentioned in lines 428-431in the discussion.

l.410: references?

These lines are no longer present in the revised manuscript.

l.411-413: Where is the evidence for the first conclusion? Not in Figure 5.

These lines have been removed in the revised manuscript.

Reviewer #3: This is a well-written and clearly presented study on the effects of environmental pH on cell size in E. coli, showing that growth in acidic pH results in smaller cells compared to neutral and basic pH growth conditions, irrespective of growth rate. Such behavior strongly points towards enhanced cell division at lower pH. The authors present compelling evidence for the enhanced accumulation of FtsN at division sites at low pH as the mechanism underlying the observed reduced cell length. The enhanced localization is dependent on the presence of FtsN's cytoplasmic FtsA-binding and periplasmic CCD domains, and can partially suppress or totally bypass the essentiality of late division proteins, including FtsI, FtsQ, and FtsK. Although the actual mechanism that triggers enhanced FtsN recruitment by low pH conditions remains unclear, this study contributes more to our understanding of the mechanistic regulation and assembly of the divisome and how extracellular factors can perturb the balance between division and cell size control. However, the manuscript requires some modifications, as indicated below.

Major comments:

1. Given the authors' previous work on the differential fitness contributions of the Class A PBPs in acidic and basic media, and the recent publication (PMID: 30504892) linking FtsN to the PBPs (which should certainly be cited), the authors need to include some discussion on how their interpretation of their data fits into the previous studies.

We have commented on this in the discussion (lines 400-406). Briefly, we suggest that FtsN coordinates the activities of the septal cell wall synthesis machinery with that of PBP1b, which has recently been suggested to function in cell wall quality control (PMID: 31904338; 30723128). Thus, midcell enrichment of FtsN in acidic media may also augment PBP1b

activity at the septum and thus direct it to the region with the highest rates of peptidoglycan synthesis. Consistent with this model, our previous work indicates that loss of PBP1b in acidic media leads to septal bulging and lysis.

2. Fig. 4D is confusing. The histogram actually shows the % of GFP-FtsN at the septum, not FtsN. The % of FtsN at the septal ring is not 0 in WT cells, as it must already be at the septum in order for them to divide. A better measure would be IFM using Anti-FtsN, or to change the Y axis to "GFP-FtsN" and to state more clearly that this is a measure of how much GFP-FtsN localizing to the septum, not total FtsN. The same is true for Fig. 5C.

We have amended the Y axes to read "% GFP-FtsN septal ring".

3. The legend for figure 5 needs to be corrected; the description of panel C is missing and the description for panel D is mislabeled.

Corrected.

4. Can the authors explain why they see stimulation of division by excess FtsN (or GFP-FtsN) whereas previously this was not seen (or excess FtsN inhibited division)? The WT (or uninduced) cell length of >4 μm seems high. Could that be why?

(Also see response to Reviewer 1). In response to this comment as well as a similar one from reviewer 1, we revisited previous studies of FtsN. Closer examination revealed that in several cases where *ftsN* overexpression inhibited growth and division, media composition was significantly more minimal than the rich conditions we used in this study. Our data indicate that while *ftsN* overexpression in rich medium (LB medium) had no detectable impact on growth rate, overexpression in minimal medium (AB medium + 0.2% glycerol) significantly curtailed cell growth (Figure S12).

5. Supplementary Figure 4. The authors attempt to show that the pH-dependent changes in cell length are due solely to FtsN by demonstrating that cells deficient in other periplasmic pH-responsive proteins show the same phenotype. However, in their previous publication (ref. 8), ΔmrcB cells did not grow in acidic media and eventually lysed. It seems that this is an inappropriate conclusion to draw from this experiment based on what the authors reported previously.

To clarify, in our previous publication (Mueller et al., eLife, 2019), we demonstrated that *ΔmrcB* failed to grow (and lyse) during growth at pH values at or below 4.8. At pH 5.5 (the condition used here), the growth defect for the *ΔmrcB* mutant is more modest (~15% defect in mass doubling time compared to the parental strain). Moreover, prior to lysis, *ΔmrcB* cells maintain normal growth rate and morphology when compared to the parental train.

6. Figures 6A and 4E; lines 321-329. Based on their truncation mutants, the authors conclude that the FtsA-binding domain and CCD domain of FtsN are both required for the cell-shortening phenotype, as well as localization to the septum. They reason that the FtsA binding domain is less important in this interaction since the truncation mutant 1-81 does not exhibit pH-dependent localization. However, this conclusion may be erroneous, as GFP-FtsN(1-55) localizes quite efficiently to the septum as reported in ref. 60. Is the GFP used here different? Or perhaps the region between 55 and 81 of FtsN inhibits its septal localization?

We thank the reviewer for drawing our attention to this puzzling discrepancy. In light of this, we have opted to test the role of the FtsA interaction interface a new way: by examining the septal localization of our GFP-FtsN(1-105)-RRKK>DDEE mutant (which is impaired for FtsA interaction) across the pH conditions. This analysis revealed, in fact, that interaction with FtsA is required for efficient localization of FtsN to the septum under each pH condition, consistent with previous reports (ref. 60; Liu et al., Mol. Microbiol., 2014). We have revised our conclusions in this section (now lines 270-282) to emphasize that the SPOR domain is not required for pH-dependent GFP-FtsN septal localization.

As for the discrepancy in localization patterns between the 1-81 and 1-55 truncation mutants, we would like to point out that the low septal localization frequency of the 1-81 truncation has been previously reported by Gerding et al., Mol. Microbiol., 2009. In that study, cells only scored positive for a GFP septal ring 6% of the time, compared to 42% for the full length FtsN and 15% for the FtsN(1-243) truncation; these figures are quite similar to our reported values (Figure 4B). In contrast, in ref. 60, upon overexpression of full length *ftsN* and the 1-55 truncation, cells score positive for a septal ring 50-70% of the time. This might suggest, as the reviewer notes, that amino acids 55-81 are indeed inhibitory. This is excellent fodder for future studies but beyond the scope of our present work.

7. The title should be reworded—“division assembly” is an ambiguous term. I suggest replacing “division assembly” with something more specific, such as “cell division”, “division septum formation”, or “cytokinesis”. I am also not a fan of using “impacts” as a verb, but realize that it is now in common usage.

The title has been modified to “pH-dependent activation of cytokinesis modulates Escherichia coli cell size”

Additional comments:

1. Figure 2: In panel A, the temperature sensitive allele of FtsA is labeled “ftsA12”; however, in the text the primary ts allele of FtsA referenced is FtsA27 (line 180), with FtsA12 being mentioned for the supplementary figure S5 (line 186).

Corrected to ftsA27.

2. Line 192: Fig. 2B is cited here, but it does not show data for ftsA27 or ftsZ84 as stated.

Corrected. Only Figure S5 is referenced here now.

3. Figure 5D. Can the authors comment on why there seems to be a 10-fold increase in growth of WT cells when depleted for FtsN at pH 8.0 compared to pH 5.5 or pH 7.0?

We thank the reviewer for drawing attention to this phenotype, which, although modest, is reproducible and consistent across all replicates. This increase in viable cells may reflect changes in activity of components of the cell division machinery at alkaline pH. However, illuminating the basis of this phenomenon is outside the scope of this study.

4. Figure 5C; lines 291-292. The authors state that the frequency of GFP-FtsN localization at the septum was similar between FtsA* and FtsL* cells, but it is not clear if they tested or showed this at each pH condition.

We thank the reviewer for pointing out this omission. We have added the GFP-FtsN septal localization frequency for *ftsA** (EAM747) and *ftsL** (EAM749) at pH 5.5, 7.0, and 8.0 to Table S4. GFP-FtsN frequency remains high across all pH conditions.

5. The "inactive state of divisome proteins in discrete complexes" (lines 400-405) model proposed is similar to that described in Krupka et al. (PMID: 28695917), who proposed that FtsA is maintained in a "locked" mini-ring state prior to divisome activation based on direct visualization of oligomeric structures of FtsA and FtsA* on membranes. That paper also proposed a positive feedback loop among early divisome proteins to activate cytokinesis. The authors should mention this as a possible mechanism, especially given that transition of FtsA from the OFF to ON state is contributing to their model.

We have added this citation and additional lines in the discussion about possible mechanisms of FtsN-mediated "activation" of FtsA (lines 408-410).

6. Lines 237-238: It looks like levels of GFP-FtsN are lower at high pH compared with neutral pH in both Fig. 3E (S8) and Fig. S6. There is also a more prominent degradation/processed band at higher pH. This may or may not be a factor in the sensitivity to high pH, and should be mentioned.

We have now mentioned the presence of the FtsN degradation product (lines 242-245) and added quantification of the degradation product in Figure S9 (formerly Figure S6). The abundance of the degradation product does not trend with septal localization of FtsN, suggesting that it is unlikely to be the driver of pH-dependent differences in cell size and division activation.

7. Line 249-250: Do the authors have any thoughts as to why cell width increases upon induction of GFP-FtsN?

We have added a line suggesting that the concentration-dependent changes in cell width may support a competition for the cell division and cell elongation machineries for a limiting pool of cell wall precursors, as proposed by other groups (lines 291-293)

8. Line 286: The original paper that reported shorter cell lengths from hypermorphic alleles of cell division genes should be cited as well (PMID: 17322202).

Citation added.

9. Line 296: Does the enhanced recruitment of FtsN to the septum cause hyperactivation of FtsA/FtsQLB, or mimic their hyperactivation through some other mechanism? This would seem to be an important distinction.

Although our study does not directly address this, recent work from several groups indicates that FtsN plays a direct role in converting FtsA and the septal cell wall synthesis machinery into

an “active” state. We have now added these citations and a discussion of their implications in the discussion section of the manuscript (lines 391-400).

Notably, Baranova et al. (Nature Microbiol., 2020) demonstrates that the cytoplasmic region of FtsN interacts with and enhances the persistence of treadmilling FtsZ and FtsA filaments in vitro, but this is abolished when the basic patch in the FtsA interaction interface is mutated (RRKK>DDEE). We find this mutant is defective for septal localization (Figure 4) and is unable to decrease size when overproduced (Figure 5).

Moreover, Yang et al. (BioRxiv, 2019) recently showed that expression of FtsN influences the dynamics of the septal cell wall synthesis enzyme FtsW. Directionally-moving FtsW molecules exist in two subpopulations that can be differentiated by particle velocity. There is significant evidence to indicate that the slower moving subpopulation represents the “active” state of FtsW. Consistent with FtsN’s involvement in the conversion of FtsW into the “active” state, overexpression of FtsN leads to an increase in the fraction of molecules moving at the slower velocity.

10. Line 302: The first report that certain alleles of FtsA including FtsA* can survive without functional FtsN should be cited here (PMID: 17542921).

Citation added.

Typos:

Line 217: should be “cells”

Corrected

Line 313: delete “in”

Corrected

Figure 2A: “fsK44” should be “ftsK44”.

Corrected
