# Supplementary material for: pH-dependent activation of cytokinesis modulates Escherichia coli cell size
Source: PLoS Genet. 2020 Mar 23;16(3):e1008685. doi: 10.1371/journal.pgen.1008685 (PMC7117782; doi:10.1371/journal.pgen.1008685)
Supplement: Attachment — Submitted filename: ResponsestoReviews_Round2.docx [file pgen.1008685.s020.docx]

Reviewer #1: The authors have responded appropriately to my prior concerns. The study is nicely done, and the manuscript is logically and clearly presented. I have only a couple of minor changes to suggest.

1. Lines 136 and 142: "evolutionary distant" should be "evolutionarily distant"

Corrected.
2. Line 223: "differences in in midcell localization" has an extra "in"

Corrected.
3. Line 233: should the figure reference be to Figure 3D, not 3E?

Corrected.
4. Also line 233: "SI Appendix, S8" should be "SI Appendix, Fig. S8" I think

Corrected.
